# Computational determination of hERG-related cardiotoxicity of drug candidates

**DOI:** 10.1186/s12859-019-2814-5

**Published:** 2019-05-29

**Authors:** Hyang-Mi Lee, Myeong-Sang Yu, Sayada Reemsha Kazmi, Seong Yun Oh, Ki-Hyeong Rhee, Myung-Ae Bae, Byung Ho Lee, Dae-Seop Shin, Kwang-Seok Oh, Hyithaek Ceong, Donghyun Lee, Dokyun Na

**Affiliations:** 1 School of integrative engineering, Chung-Ang University, Seoul, Republic of Korea; 2 College of Industrial Sciences, Kongju National University, Yesan, Republic of Korea; 3 Bio-based Technology Research Center, Korea Research Institute of Chemical Technology, 141 Gajeong-ro, Yuseong, Daejeon, 34114 Republic of Korea; 4 Information-based Drug Research Center, Korea Research Institute of Chemical Technology, 141 Gajeong-ro, Yuseong, Daejeon, 34114 Republic of Korea; 5 Department of Multimedia, Chonnam National University, Yeosu, Republic of Korea

**Keywords:** In silico model, Machine learning, hERG-related cardiotoxicity, Drug discovery

## Abstract

**Background:**

Drug candidates often cause an unwanted blockage of the potassium ion channel of the human ether-a-go-go-related gene (hERG). The blockage leads to long QT syndrome (LQTS), which is a severe life-threatening cardiac side effect. Therefore, a virtual screening method to predict drug-induced hERG-related cardiotoxicity could facilitate drug discovery by filtering out toxic drug candidates.

**Result:**

In this study, we generated a reliable hERG-related cardiotoxicity dataset composed of 2130 compounds, which were carried out under constant conditions. Based on our dataset, we developed a computational hERG-related cardiotoxicity prediction model. The neural network model achieved an area under the receiver operating characteristic curve (AUC) of 0.764, with an accuracy of 90.1%, a Matthews correlation coefficient (MCC) of 0.368, a sensitivity of 0.321, and a specificity of 0.967, when ten-fold cross-validation was performed. The model was further evaluated using ten drug compounds tested on guinea pigs and showed an accuracy of 80.0%, an MCC of 0.655, a sensitivity of 0.600, and a specificity of 1.000, which were better than the performances of existing hERG-toxicity prediction models.

**Conclusion:**

The neural network model can predict hERG-related cardiotoxicity of chemical compounds with a high accuracy. Therefore, the model can be applied to virtual high-throughput screening for drug candidates that do not cause cardiotoxicity. The prediction tool is available as a web-tool at http://ssbio.cau.ac.kr/CardPred.

## Background

Many drug candidates are withdrawn owing to unexpected side effects. Therefore, it is a major challenge to screen out potential toxic compounds in the drug discovery process. Cardiac toxicity is one of the side effects and a major cause of drug withdrawals in drug discovery. A representative mechanism of cardiotoxicity involves the binding of compounds to the cardiac potassium channel encoded by the human *ether-a-go-go*-related gene (hERG), which results in long QT syndrome (LQTS) and eventually leads to fatal ventricular arrhythmias and sudden death [1, 2]. Recently, many drugs, such as terfenadine, cisapride, astemizole, sertindole, thioridazine, and grepafloxacin, were withdrawn from the market owing to undesired cardiotoxicity effects [3]. The development of an accurate prediction model for hERG channel blockers is, therefore, essential in the early stage of drug development.

Experimental high-throughput screening methods have been developed [4], but experimental methods for drug-induced cardiotoxicity are time-consuming and costly. Thus, it is necessary to develop a computational approach to accelerate drug discovery. In recent years, several ligand-based in silico models have been developed to predict drug-hERG interactions based on the pharmacophore, quantitative structure-activity relationship (QSAR), and classification models [5–8].

The first pharmacophore model was developed based on steric and electronic features associated with the biological effects on hERG binding affinity using 15 compounds by Ekins et al. [9]. Because conventional pharmacophore models were generally developed using small training datasets of fewer than 500 [10, 11], their applicability was highly limited. Thus, ensemble models integrating diverse pharmacophore methods have also been developed for a better prediction of the hERG binding affinity [5, 12].

Three-dimensional (3D)-QSAR models based on 3D structure information, such as the molecular interaction fields, have been developed to predict the correlation between the 3D structure information and hERG binding affinity by regression analysis. Two representative methods used for 3D-QSAR modelling were the comparative molecular field analysis (CoMFA) [13] and grid-independent descriptors (GRIND) [14]. Both 3D-QSAR models exhibited a high performance in predicting the binding affinity for most compounds that were not lipophilic compounds [13, 15].

Classification models for toxicity prediction have been developed using a set of physicochemical descriptors. To improve prediction performance, various machine learning algorithms have been employed, including the support vector machine (SVM), naïve Bayes, decision tree, random forest, and k-nearest neighbors (kNN) [16–19]. The machine learning algorithms have facilitated the advancement of prediction model development, but the inclusion of inconsistent experimental data included in training datasets damps the development of accurate prediction models [20]. Available hERG toxicity datasets were compiled from the literature in which experiments were conducted under different conditions and the definition of toxicity was also different. To our knowledge, there are no large hERG toxicity datasets obtained from a single study. Recently, Czodrowski et al. developed a hERG toxicity prediction model using a large dataset containing 4415 compounds extracted from the ChEMBL database [20]; however, the model showed a low AUC value because of the inconsistency of the database. Because the hERG toxicity database was compiled from the literature, it included many inconsistent experimental data.

For this study, we generated a large experimental dataset of hERG assay results from 2130 chemicals, which were carried out under the same conditions. Similar to the ChEMBL hERG toxicity database, publicly available datasets were generally collected from the literature and may contain many inconsistent data. Such inconsistency may lead to inaccurate computational models. Our dataset was used to train machine learning models (linear regression, ridge regression, logistic regression, naïve Bayes, neural network, and random forest), and it was found that the model using the neural network showed a higher Matthews correlation coefficient (MCC) of 0.368, than the other models. In addition, when the neural network model was further evaluated using a test dataset of ten drug compounds obtained from in vivo experiments in this study, the model showed a high accuracy of 80% (MCC of 0.655). Therefore, the developed hERG-toxicity prediction model can be utilized as a virtual screening tool for the identification of the cardiotoxicity of drug candidates in the early stage of drug discovery.

## Materials and methods

### Binding assay for hERG based on fluorescence polarization

The fluorescence polarization (FP)-based binding assay for hERG was measured according to the protocol of the Predictor™ hERG FP kit (Thermo Fisher Scientific, Inc., Rockford, IL, USA). The membrane fraction containing the hERG channel protein (Predictor™ hERG membrane) and tracer (Predictor™ hERG tracer red) was prepared with dilution in the binding buffer provided by the manufacturer. The binding assay was conducted in a final volume of 20 μL with a 10 μL membrane, 5 μL of a 4 nM tracer, and 5 μL of test compounds. The assays were conducted in 384 well black flat-bottom microplates (Corning Life Sciences, Lowell, MA, USA). After incubation for 4 h at room temperature, the FP was determined using a multimode reader (Infinite M1000PRO; Tecan, Mannedorf, Switzerland) in the FP detection mode, with excitation and emission filters of 535 and 590 nm, respectively.

### In vivo experimental procedures and recordings of electrocardiography

In this study, guinea pigs were used and fasted for 18 h prior to the experimental procedures. The animals were anesthetized with sodium pentobarbital (60 mg/kg, i.p.), followed by artificial respiration using a rodent ventilator (60 strokes/min, 1 ml/100 g BW). The animals were placed on a heat pad with circulating water at a temperature of 37 °C. A catheter was inserted into the jugular vein for drug administration, and electrocardiography (ECG) pin electrodes were positioned for the standard limb lead and chest lead configurations. All the animals were allowed to stabilize for 20 min after being instrumented, prior to drug administration. When the heart rate of each animal was constant, the lowest concentration of the drug was administered for 1 min through the jugular vein. After 10 min, the test drug at the following concentration was administered according to the cumulative method. The QRS complex and the PR, QT, PRC, and QRc intervals were measured with the ECG measurement yields, in addition to the heart rate, for the evaluation of the cardiac function. The values were expressed as the mean and standard deviations of each group. The data were analyzed using the one-way analysis of variance (ANOVA) followed by Dunnett’s test, to verify the significant differences between the groups.

### Data preparation

The hERG toxicities of 2130 compounds were measured as IC_50_ values. Compounds with IC_50_ < 10 μM were classified as toxic and the other compounds were classified as nontoxic [19]. Consequently, 221 compounds (10.38%) were identified as hERG-toxic, and 1909 compounds (89.62%) were identified as nontoxic. The toxicities of ten drug compounds obtained from in vivo experiments, which were not included in the 2130 compounds, were used for testing our developed model.

### Descriptor calculation

The compounds from the hERG toxicity assays were expressed in the simplified molecular-input line-entry system (SMILES) format [21], and the SMILES were used for the DRAGON software (version 7.0.10) to calculate their physicochemical descriptors and fingerprints (2432 nonconstant molecular descriptors) [22]. In addition, extended connectivity fingerprints (ECFPs) were also generated [23] with a maximum diameter parameter of 4 and length parameter of 1024. Thus, in this study, 3456 molecular features were used for the training of the learning models.

### Feature correlation calculation and feature selection

To reduce the number of features in developing the prediction models, 3456 features were ranked in order of their correlation with toxicity. The phi coefficient was calculated for binary features [24], and the point-biserial correlation coefficient was calculated for continuous features [25].

To calculate the point-biserial correlation coefficient, the dataset was divided into toxic and nontoxic molecules. The point-biserial correlation coefficient (*r*_*pb*_) was calculated as follows:

1 $$ {\displaystyle \begin{array}{c}{r}_{pb}=\frac{M_{toxic}-{M}_{nontoxic}}{s_n}\sqrt{\frac{n_{toxic}\times {n}_{nontoxic}}{n^2}}\\ {}\mathrm{where}\ {s}_n=\sqrt{\frac{1}{n}\sum \limits_{i=1}^n{\left({X}_i-\overline{X}\right)}^2,}\end{array}} $$

*M*_*toxic*_ and *M*_*nontoxic*_ denote the mean feature values of the toxic and nontoxic compounds, respectively. *n*_*toxic*_ and *n*_*nontoxic*_ denote the numbers of toxic and nontoxic compounds, respectively, and *n* is the total number of molecules. *s*_*n*_ denotes the standard deviation of the feature. *X*_*i*_ represents each feature value and $\overline{X}$ denotes the mean value of all the feature values.

The phi coefficient (**∅)** was calculated as below:

2 $$ \mathbf{\varnothing}=\frac{n_{toxic\bullet 1}\times {n}_{nonto xic\bullet 0}-{n}_{toxic\bullet 0}\times {n}_{nonto xic\bullet 1}}{\sqrt{\left({n}_{toxic\bullet 1}+{n}_{toxic\bullet 0}\right)\left({n}_{toxic\bullet 1}+{n}_{nonto xic\bullet 1}\right)\left({n}_{nonto\mathrm{x} ic\bullet 1}+{n}_{nonto xic\bullet 0}\right)\left({n}_{toxic\bullet 0}+{n}_{nonto xic\bullet 0}\right)}} $$

where *n*_*toxic* ∙ 1_ and *n*_*toxic* ∙ 0_ denote the number of features of toxic compounds, which are 1 and 0, respectively. *n*_*nontoxic* ∙ 1_ and *n*_*nontoxic* ∙ 0_ denote the number of features of nontoxic compounds, which are 1 and 0, respectively.

### Models

Six machine learning algorithms were used to construct the hERG toxicity prediction models. The linear regression is a simple regression algorithm that models the linear relationship between a dependent variable and multiple explanatory variables [26]. The ridge regression is an advanced linear regression model that introduces a ridge regularization method for the optimization of the model [27]. The logistic regression is a regression algorithm that models a logistic relationship, which can be used for binary classification [28]. A naïve Bayes is a probabilistic classification model based on the Bayesian theorem and the naïve independency between features [29]. A random forest is an ensemble model that constructs multiple decision trees and combines them to derive a merged result [30]. A neural network is a machine learning model that refers to a network structure composed of artificial neurons and nodes, which can optimize the network to recognize patterns of input data [31]. These algorithms were implemented in the Orange 3 Python machine learning package, and, in this study, Orange 3 was used to develop the hERG toxicity prediction models [32].

### Performance evaluation

The six models trained with our dataset were evaluated by ten-fold cross-validation. In this process, the optimal number of features was also determined by the area under the receiver operating characteristic curve (AUC). Because the dataset was biased to nontoxic compounds, we also calculated the MCC that is an accuracy measure for unbalanced datasets. After the cross-validation and feature number optimization, the best model was determined. This model was further evaluated with ten drug compounds that were not included in the training dataset and were tested in vivo on guinea pigs to assess the applicability of our model developed using in vitro data to in vivo toxicity. The performance of our model was compared with other hERG prediction tools, the Pred-hERG 4.1 [6] and OCHEM Predictor [33].

## Results and discussion

### Model construction

**Fig. 1:**
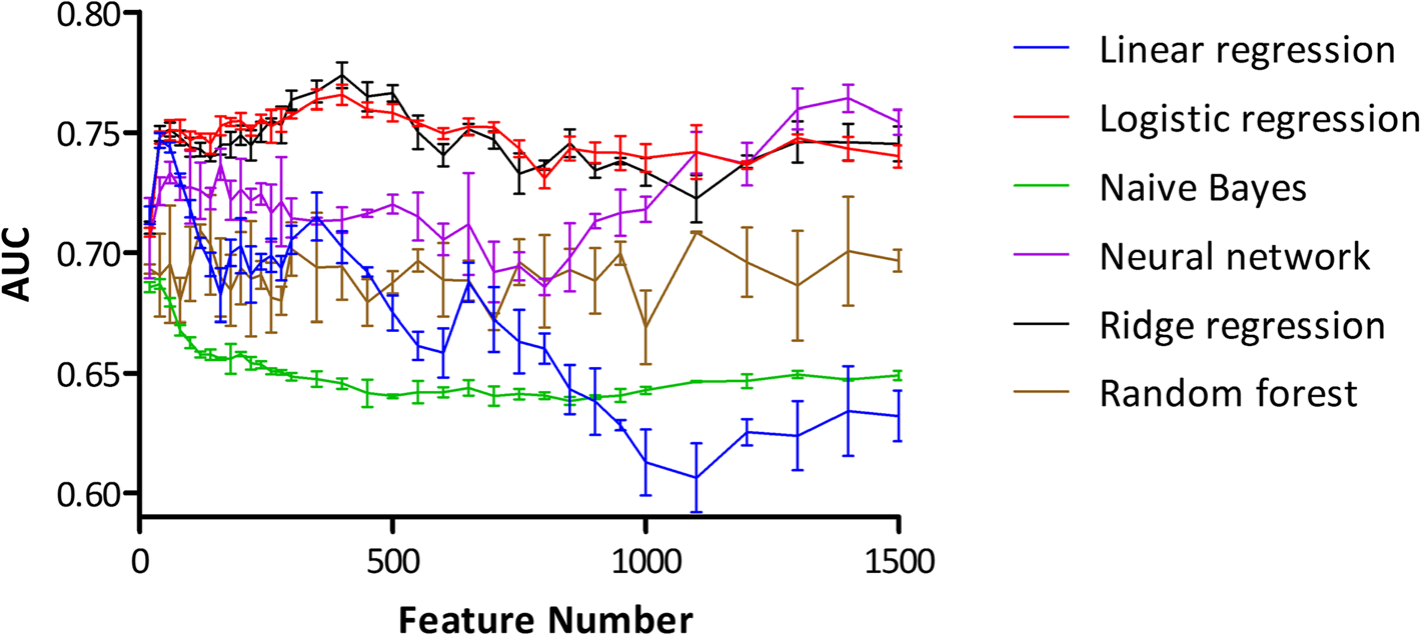
AUC with respect to feature number: AUC values of the six models were measured by a ten-fold cross-validation with respect to feature number

Correlation coefficients between the features and toxicity were calculated and the top-ranked features were used to train models. The top 20 features are listed in Table 1. Computational hERG prediction models were trained using six different machine learning algorithms with a different number of top features. The six algorithms were linear regression, ridge regression, logistic regression, artificial neural network, naïve Bayes, and random forest. Their ten-fold cross-validation results and respective optimal feature numbers are shown in Fig. 1 and Table 2. Of the six models, those developed based on the neural network (AUC = 0.764, feature = 1400), ridge regression (AUC = 0.774, feature = 400), and logistic regression (AUC = 0.764, feature = 350) showed better performances than those of the other models. Because the performances of the three models were comparable, they were further optimized to determine the best model.

**Table 1:** Top 20 features with a high correlation

Descriptor	Coeff.	Description
nRNR2	0.229	Number of tertiary amines (aliphatic)
Wap	0.215	All-path Wiener index
F02[C-C]	0.212	Frequency of C - C at topological distance 2
F03[C-C]	0.212	Frequency of C - C at topological distance 3
nC	0.211	Number of carbon atoms
F04[C-C]	0.210	Frequency of C - C at topological distance 4
D/Dtr06	0.208	Distance/detour ring index of order 6
ATSC5v	0.207	Centred Broto–Moreau autocorrelation of lag 5 (weighted by van der Waals volume)
F01[C-C]	0.205	Frequency of C - C at topological distance 1
SpDiam_Dt	0.205	Spectral diameter from detour matrix
SpAD_Dt	0.204	Spectral absolute deviation from detour matrix
SpPos_Dt	0.204	Spectral positive sum from detour matrix
N-068	0.203	Atom-centered fragment: Al3-N
Wi_Dt	0.203	Wiener-like index from detour matrix (detour index)
SpMax_Dt	0.203	Leading eigenvalue from detour matrix
TI1_L	0.203	First Mohar index from Laplace matrix
H_Dz(p)	0.202	Harary-like index from Barysz matrix (weighted by atomic number)
IDET	0.202	Total information content on the distance equality
F10[C-C]	0.202	Frequency of C - C at topological distance 10
nR06	0.201	Number of six-membered rings

**Table 2:** Performance (AUC) results of six machine learning methods

Algorithm	Optimal number of features	AUC
Linear regression	40	0.747
Logistic regression	350	0.764
Ridge regression	400	0.774
Neural network	1400	0.764
Naïve Bayes	40	0.687
Random forest	120	0.709

### Model optimization

To select the best model, we optimized the threshold values of the three selected models, which discriminated toxic and nontoxic groups. The best threshold values that showed the highest MCC are listed in Table 3. MCC is an accuracy measure for highly unbalanced datasets. Of the three models, the neural network model showed the best performance, with an accuracy of 90.1%, an MCC of 0.368, and a positive predictive value (PPV) of 0.542 after threshold optimization. The low sensitivity and high specificity of the neural network model were due to its high threshold value, but the high threshold improved its performance expressed as MCC. Consequently, the toxicity prediction model based on the neural network was selected for further evaluation.

**Table 3:** Performance results of the top three models with optimized thresholds

Algorithm	Threshold	Accuracy	MCC	Sensitivity	Specificity	PPV^a^
Logistic regression	0.57	0.814	0.307	0.557	0.844	0.292
Neural network	0.82	0.901	0.368	0.321	0.967	0.542
Ridge regression	0.64	0.864	0.332	0.448	0.912	0.371

^a^PPV: Positive predictive value is defined as the number of true positives/(the number of true positives + the number of false positives)

### Test of the constructed model on in vivo data

The optimized model was further tested on ten known drug molecules, whose cardiotoxicities were measured in vivo using guinea pigs. In vitro experiments are simpler and less expensive than in vivo experiments, hence, they can be carried out at a larger scale. However, owing to the complex physiology of in vivo systems, in vitro experimental results are often inconsistent with in vivo results. Thus, we further evaluated the applicability of our model that was trained using in vitro data to the in vivo toxicity. The prediction results of the test compounds are shown in Tables 4 and 5. Our model showed an overall accuracy of 80.0%, an MCC of 0.655, a sensitivity of 0.600, a specificity of 1.000, and a PPV of 1.000. This high performance indicates that our model could also be utilized to predict in vivo cardiotoxicity.

**Table 4:** Prediction results of ten drug compounds

Name	In vivo result		Prediction		
		Our model	Pred-hERG binary	Pred-hERG multiclass	OCHEM Predictor^a^
Haloperidol	Toxic	Toxic	Toxic	Nontoxic	Nontoxic
Cimetidine	Nontoxic	Nontoxic	Toxic	Nontoxic	Nontoxic
Disopyramide	Toxic	Toxic	Nontoxic	Nontoxic	Nontoxic
Quinnidine	Toxic	Nontoxic	Toxic	Nontoxic	Toxic
Terazosin	Nontoxic	Nontoxic	Toxic	Nontoxic	Nontoxic
Spironolactone	Nontoxic	Nontoxic	Toxic	Nontoxic	Nontoxic
Sotalol	Toxic	Nontoxic	Nontoxic	Nontoxic	Nontoxic
Cefazoline	Nontoxic	Nontoxic	Toxic	Nontoxic	Nontoxic
Chloropromazine	Toxic	Toxic	Toxic	Toxic	Nontoxic
Loratadine	Nontoxic	Nontoxic	Toxic	Nontoxic	Nontoxic

^a^Consensus II in the predictor was used

**Table 5:** Performance comparison on the in vivo test dataset

Models	Accuracy	MCC	Sensitivity	Specificity
Our model	0.800	0.655	0.600	1.000
Pred-hERG binary	0.300	−0.500	0.600	0.000
Pred-hERG multiclass	0.600	0.333	0.200	1.000
OCHEM Predictor	0.600	0.333	0.200	1.000

Several computational methods have been reported for the prediction of hERG toxicity (Pred-hERG and OCHEM Predictor). We compared the performance of our model with previous methods; the prediction results of other methods are also listed in Table 5. The Pred-hERG model is a web-tool based on the statistical QSAR model of hERG channel blockers. OCHEM is also a web-tool based on eight associative neural network models. The prediction results of the ten test drug compounds using the previous methods, and their overall performances are listed in Tables 4 and 5, respectively. Pred-hERG has two models: binary and multiclass. The Pred-hERG binary model decides whether a query compound is a hERG-blocker or nonblocker. The Pred-hERG multiclass model determines the group in which a query compound belongs: nonblockers, weak/moderate blockers, or strong blockers. In this study, we considered weak/moderate and strong blockers as hERG-toxic. The binary model of the Pred-hERG predicted eight out of ten compounds as toxic molecules with an accuracy of 30%. Whereas the multiclass model of the Pred-hERG predicted nine out of ten compounds as nontoxic with an accuracy of 60%. Their MCC values were − 0.500 and 0.333, respectively. Similar to the multiclass model of the Pred-hERG, the OCHEM Predictor predicted nine out of ten compounds as nontoxic. Its accuracy and MCC were 60% and 0.333, respectively. The three previous models made biased predictions, resulting in a very low sensitivity or very low specificity (Table 5). Our model correctly predicted eight out of ten compounds with an accuracy of 80% and an MCC of 0.655, which indicates that our model outperforms other methods and would be useful for the prediction of the in vivo cardiotoxicity of drug candidates. It can also be used for virtual screening in drug discovery.

### Additional comparison with previous models

Because in vivo cardiotoxicity assays require animal experiments, it is difficult to obtain a large number of in vivo data. Performance comparison with only ten compounds was not fair, so we evaluated the performances of previous methods using the training dataset containing 2130 compounds obtained from in vitro experiments. For a fair comparison, we divided the dataset into training (90%) and test (10%) datasets; the training data was used to build our model and the remaining test dataset was used to evaluate the performances of our model, the Pred-hERG, and OCHEM Predictor. The evaluation was iterated ten times, and their averages were calculated (Table 6). The MCC values of the previous models were lower than that of our model. Specifically, the Pred-hERG binary model showed an MCC of − 0.034, a sensitivity of 0.912, and a specificity of 0.061, indicating that this model classified most query molecules as toxic and had many false positives. This high number of false positives for the Pred-hERG binary model were also shown on the test dataset (Tables 4 and 5). On the contrary, the Pred-hERG multiclass and OCHEM Predictor showed a low sensitivity and a high specificity, indicating that they classified most query molecules as nontoxic. Because the dataset was highly unbalanced to negative (nontoxic) data, the biased predictions of the Pred-hERG multiclass and OCHEM Predictor to the nontoxic class increased the accuracy to 90.2 and 88.5% and decreased their MCCs to 0.218 and 0.133, respectively. Consequently, our model consistently showed a better performance for the small test dataset as well as on the training dataset.

**Table 6:** Performance comparison on the in vitro dataset

Models	Accuracy	MCC	Sensitivity	Specificity
Our model	0.901	0.368	0.321	0.967
Pred-hERG binary	0.15	−0.034	0.912	0.061
Pred-hERG multiclass	0.902	0.218	0.075	0.999
OCHEM Predictor	0.885	0.133	0.099	0.978

## Conclusion

In this study, we aimed at producing a reliable hERG toxicity dataset and then at developing a better performing cardiotoxicity prediction model. Computational models are highly dependent on the reliability of datasets; however, the collected datasets from the literature may include inconsistent experimental results. We generated our own consistent dataset to build a model; the developed prediction model using our dataset outperformed the other hERG prediction tools. Our model can be useful for the virtual screening for potential drug candidates that do not cause cardiotoxicity and would facilitate the advancement of in silico drug discovery. However, in this study, new features and new machine learning methods were not introduced, so there is scope to improve our model further if new features specialized for describing the cardiotoxicity of molecules are included or new machine learning algorithms are used that efficiently and effectively classify molecules using the features.

## Abbreviations

AUC: Area under ROC curve
ECFP: Extended connectivity fingerprint
hERG: Human ether-a-go-go-related gene
LQTS: Long QT syndrome
MCC: Matthews correlation coefficient
PPV: Positive predictive value
SEN: Sensitivity
SMILES: Simplified molecular-input line-entry system
SPE: Specificity
